# Supplementary material for: Baseline Values of Circulating IL-6 and TGF-β Might Identify Patients with HNSCC Who Do Not Benefit from Nivolumab Treatment
Source: Cancers (Basel). 2023 Nov 2;15(21):5257. doi: 10.3390/cancers15215257 (PMC10649732; doi:10.3390/cancers15215257)
Supplement: Supplementary file 1 [file cancers-15-05257-s001.zip › cancers-2672953-supplementary.pdf]

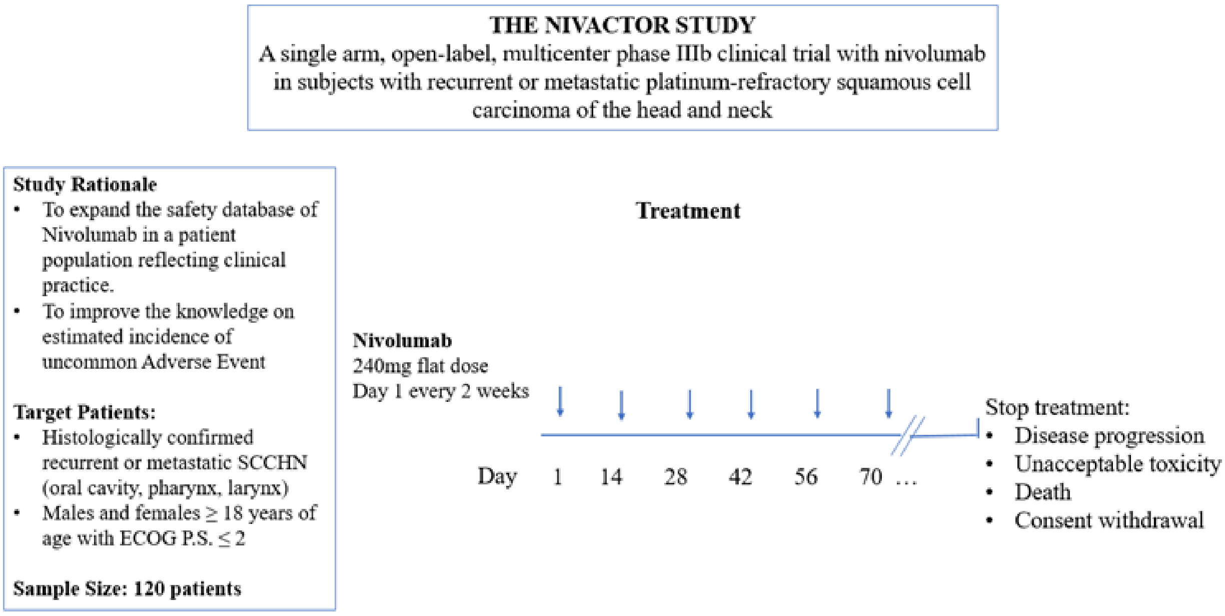

Figure S1. Representation of study design of NIVACTOR trials.

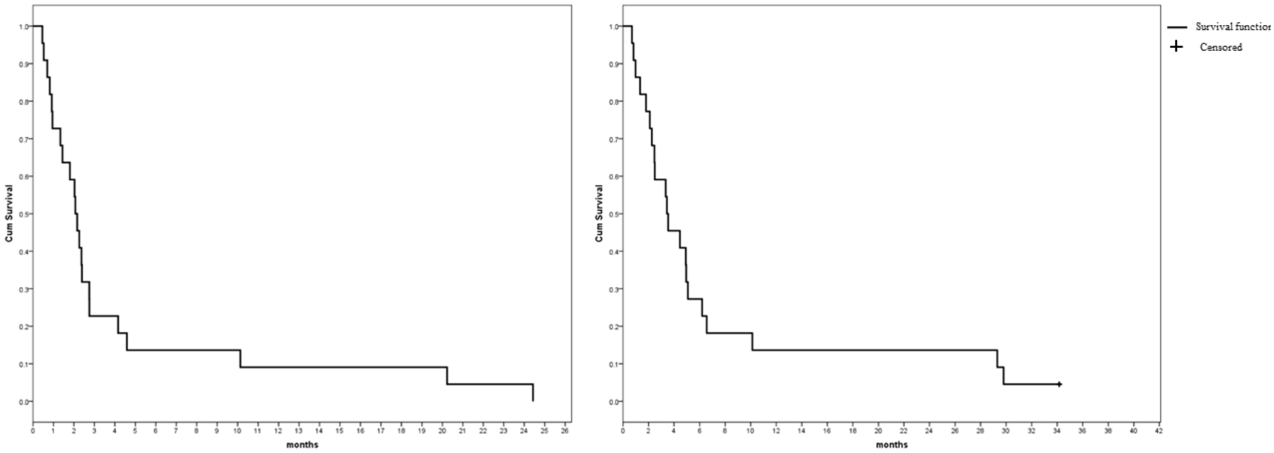

Figure S2. Kaplan Meier curves for PFS (left) and OS (right) for patient's population.

Area Under the Curve

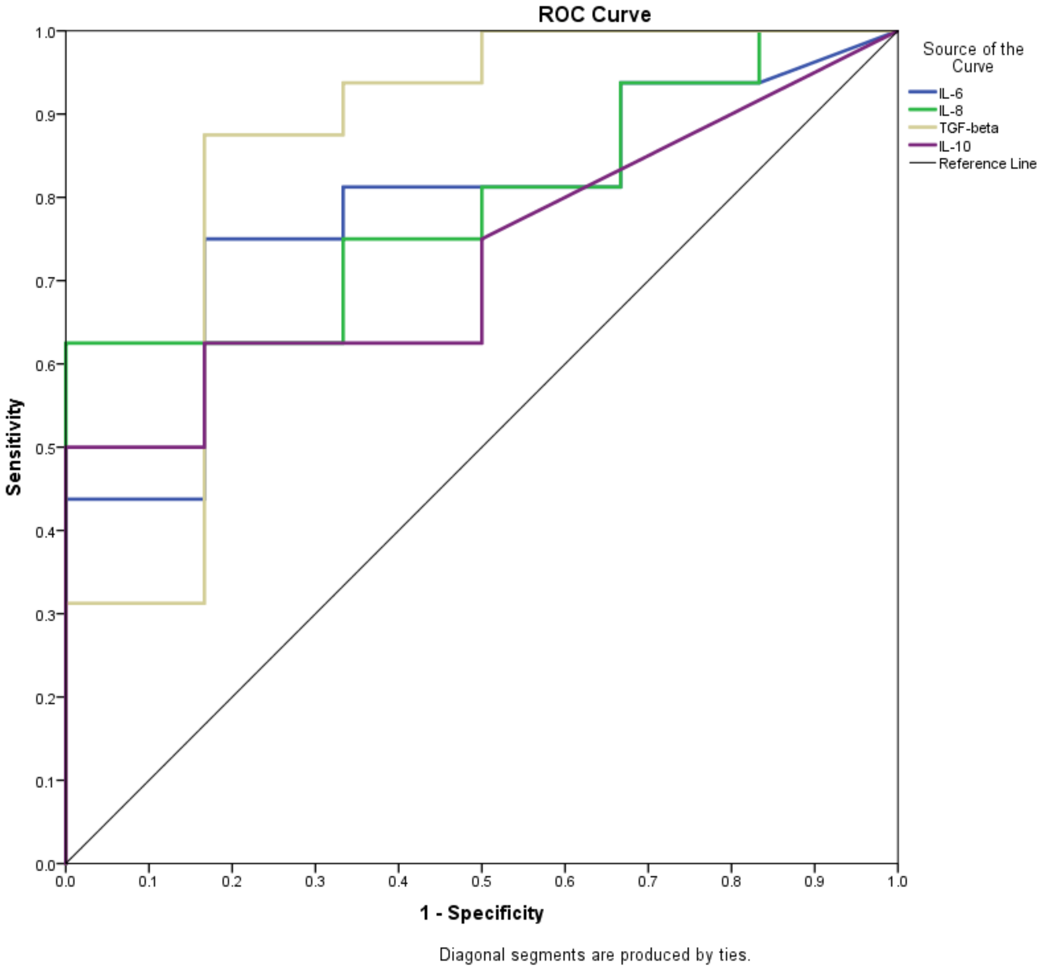

Test Result Variable(s)	Area	Std. Error[a]	Asymptotic Sig.[b]	Asymptotic 95% Confidence Interval	
				Lower Bound	Upper Bound
TGF-β	.854	.108	.012	.642	1.000
IL-6	.786	.103	.043	.585	.988
IL-8	.792	.097	.039	.602	.981
IL-10	.729	.108	.105	.518	.941

a. Under the nonparametric assumption

b. Null hypothesis: true area = 0.5

Figure S3. ROC curve and table of variables specific to identify Group 1 from Group 2.

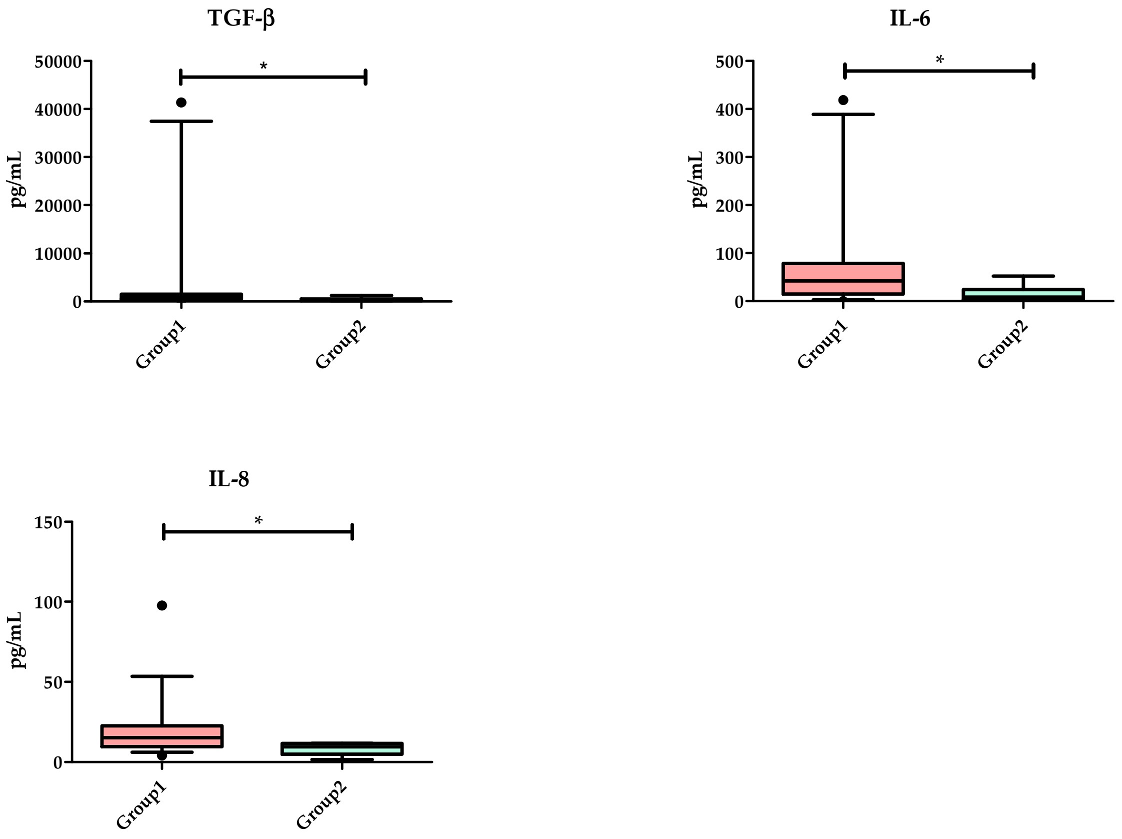

Figure S4. Distribution of TGF-β, IL-6 and IL-8 between Group1 and Group2. * $p<0.05$, ** $p<0.01$.

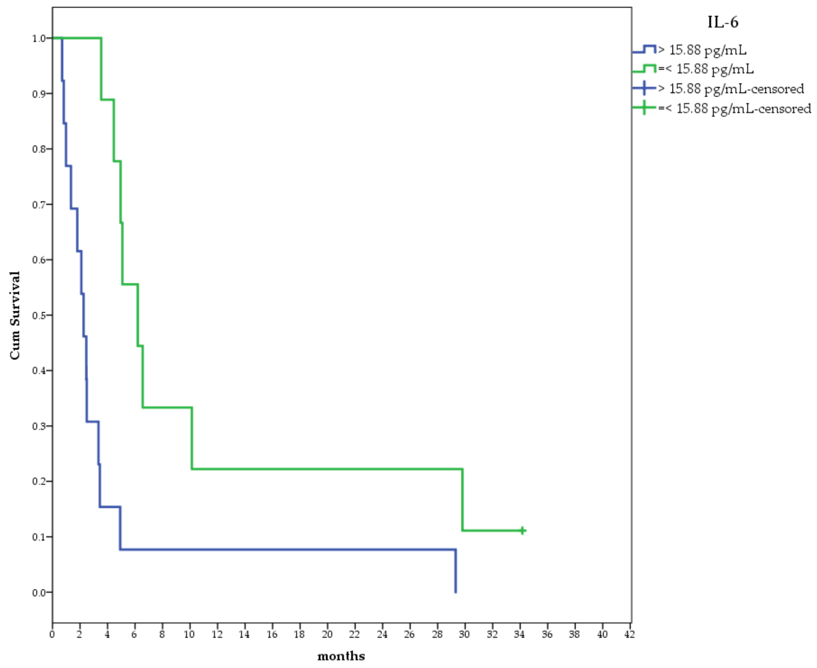
IL-6
> 15.88 pg/mL
=< 15.88 pg/mL
> 15.88 pg/mL-censored
=< 15.88 pg/mL-censored
Cum Survival
months

a

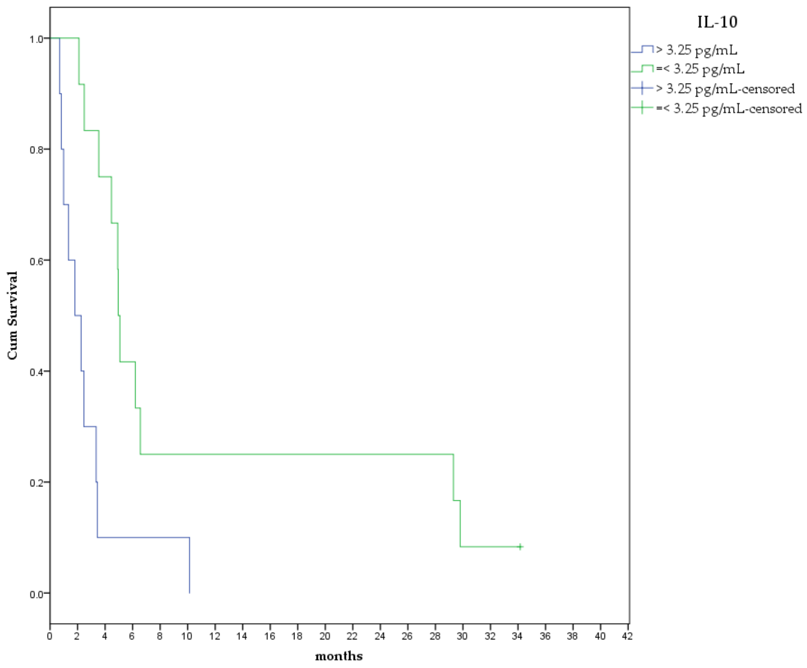
IL-10
> 3.25 pg/mL
=< 3.25 pg/mL
> 3.25 pg/mL-censored
=< 3.25 pg/mL-censored
Cum Survival
months

b

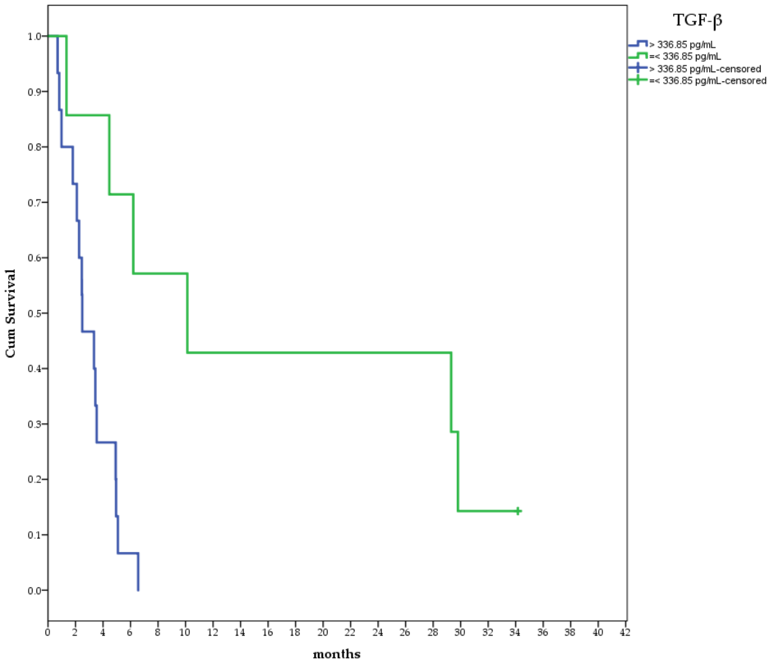

c

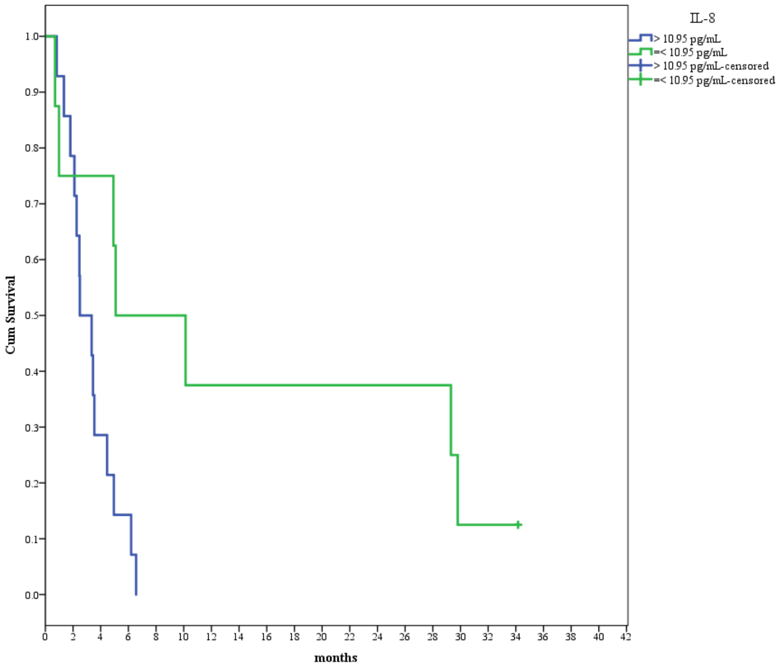

d

Figure S5. Kaplan Meier for a) IL-6, b) IL-10, c) TGF-β and d) IL-8.

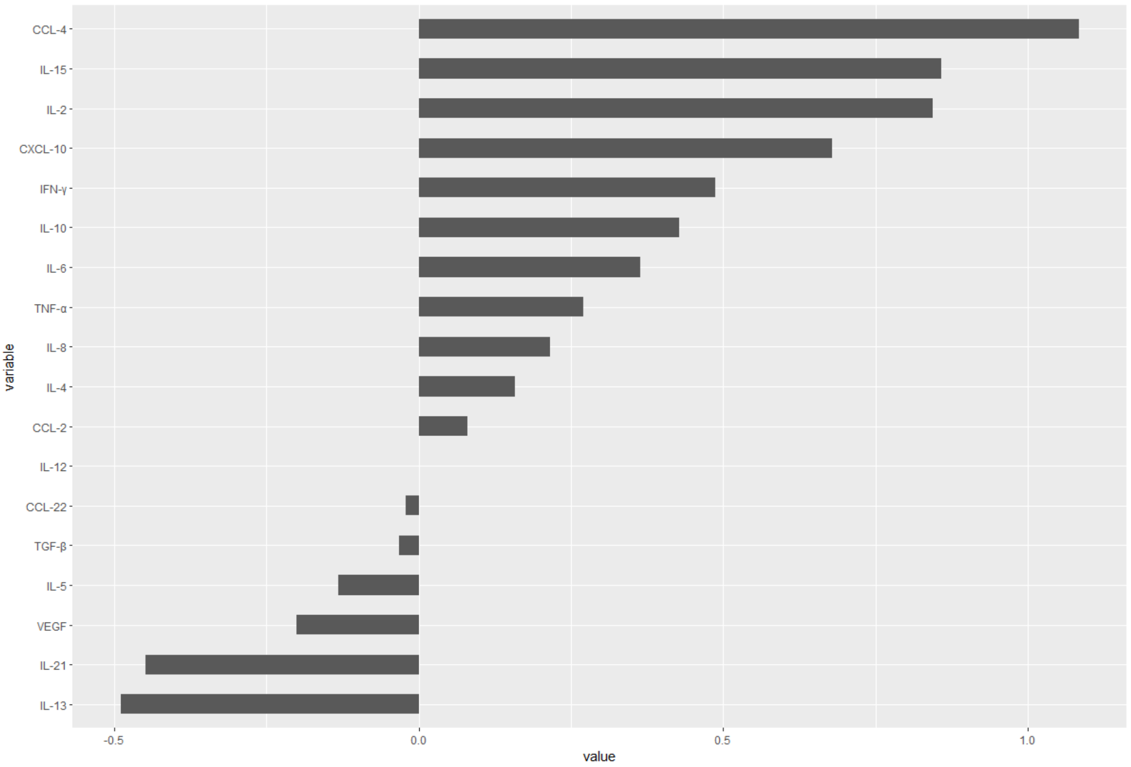

Figure S6. Longitudinal changes of 18 cytokines from T0 set to 0. Values are expressed as ratio calculated as follow: $(T1-T0)*T0^{-1}$.

Table S1. Cytokine value for each patients at T0 and T1.

			IL-21		CCL-2		CCL-4		CCL-22		CXCL-10		VEGF		IFN-γ		TGF-β		TNF-α		IL-2		IL-4		IL-5		IL-6		IL-8		IL-10		IL-12		IL-13		IL-15	
**Age**	**PFS**	**OS**	**t0**	**t1**	**t0**	**t1**	**t0**	**t1**	**t0**	**t1**	**t0**	**t1**	**t0**	**t1**	**t0**	**t1**	**t0**	**t1**	**t0**	**t1**	**t0**	**t1**	**t0**	**t1**	**t0**	**t1**	**t0**	**t1**	**t0**	**t1**	**t0**	**t1**	**t0**	**t1**	**t0**	**t1**	**t0**	**t1**
62	2.39	4.46	3.06	2.90	32.50	174.21	37.70	43.80	160.00	221.00	105.00	187.00	43.24	42.34	8.94	12.19	233.70	147.04	0.00	0.65	0.49	0.00	0.00	0.00	1.10	0.64	14.73	45.36	14.40	24.30	0.00	0.00	1.42	1.64	572.17	0.00	0.00	0.00
75	1.44	29.31	3.62	5.83	21.69	44.18	0.43	20.60	47.20	224.00	26.70	734.00	35.62	64.89	4.79	0.02	71.51	223.48	0.95	0.80	3.20	0.00	0.00	0.00	0.00	0.01	52.33	11.75	1.50	5.76	0.00	0.00	3.35	2.70	141.45	185.38	4.82	0.17
57	2.33	6.20	3.54	3.54	115.70	35.43	22.00	17.00	236.00	246.00	86.80	142.20	30.88	30.88	0.00	18.78	153.07	264.50	8.41	1.98	0.00	6.29	0.00	0.00	0.00	0.00	0.00	5.77	11.78	8.64	0.00	0.00	3.69	2.44	148.56	110.91	1.61	0.26
56	2.75	3.54	3.14	3.30	45.63	60.20	39.60	40.60	429.00	129.00	189.00	600.00	57.62	48.31	1.91	7.58	680.50	407.64	0.00	0.00	2.42	0.00	0.00	0.00	0.10	0.01	5.22	24.51	19.00	16.06	0.00	0.00	9.61	4.54	0.00	12.24	0.00	3.07
60	4.59	4.95	4.01	0.00	411.00	58.49	21.00	34.80	252.00	345.00	67.80	146.00	32.43	41.05	3.71	0.00	862.12	327.70	35.00	0.00	0.00	0.00	0.00	0.00	0.00	0.56	3.74	74.75	14.80	16.50	0.00	6.95	5.54	12.46	524.02	21.11	3.77	13.22
84	4.16	5.08	3.54	3.54	21.06	335.00	37.40	48.10	834.00	284.00	456.00	347.00	23.72	150.42	1.57	3.20	1101.99	684.20	0.00	3.47	0.00	1.65	0.00	0.00	0.00	0.00	4.17	40.90	6.84	2.58	3.11	5.73	4.37	4.65	0.00	266.00	2.67	2.63
69	20.23	29.80	4.41	0.00	98.84	42.61	57.40	226.00	494.00	1057.00	177.00	632.00	168.27	51.88	0.00	3.47	242.32	370.63	5.68	0.00	0.00	6.38	0.00	0.00	0.00	0.88	3.26	53.93	10.80	30.80	0.00	5.07	8.78	4.38	0.00	0.00	2.03	45.90
64	2.07	3.44	18.20	0.00	107.20	16.57	41.20	63.40	488.00	201.00	544.00	845.00	218.85	3.93	0.00	0.00	1092.31	948.00	5.96	0.00	0.00	9.85	0.00	0.00	0.67	1.22	71.77	17.78	23.00	25.80	10.06	5.64	3.05	2.44	24.00	0.00	1.62	4.93
77	2.26	2.26	51.11	/	35.87	/	49.50	/	167.00	/	80.60	/	12.54	/	0.00	/	957.26	/	0.00	/	0.00	/	0.00	/	1.47	/	330.24	/	15.80	/	12.97	/	4.64	/	0.00	/	11.26	/
63	1.34	1.34	0.00	/	37.85	/	71.90	/	287.00	/	555.00	/	16.11	/	0.00	/	262.30	/	0.00	/	0.66	/	0.00	/	0.91	/	65.42	/	18.90	/	12.22	/	0.00	/	0.00	/	7.08	/
75	0.82	0.82	0.00	/	35.72	/	31.90	/	230.00	/	324.00	/	50.24	/	2.28	/	604.31	/	0.00	/	0.00	/	0.00	/	1.32	/	418.60	/	11.10	/	3.38	/	0.00	/	0.00	/	20.00	/
70	24.43	34.16	1041.64	621.40	125.77	114.00	64.80	48.00	1212.00	976.00	586.00	605.00	2.35	8.12	0.00	2.44	289.98	491.80	0.00	6.27	3.77	0.27	0.00	0.17	0.54	0.61	8.56	1.49	8.10	7.29	2.82	3.55	0.00	0.64	0.00	1.41	4.68	2.49
78	0.69	0.69	0.00	/	68.17	/	18.90	/	118.00	/	194.00	/	163.74	/	0.00	/	683.44	/	1.56	/	1.76	/	0.00		0.56	/	16.47	/	7.00	/	10.15	/	22.26	/	0.00	/	5.73	/
71	0.95	2.46	0.00	0.00	71.60	50.00	20.70	70.90	452.00	730.00	1342.00	2960.00	51.77	6.51	0.00	0.00	1241.74	949.37	0.72	30.57	3.77	1.00	0.00	0.11	0.56	0.65	22.32	1.28	11.20	38.40	10.15	4.86	2.09	1.51	0.00	1.19	2.12	3.22
71	2.00	4.92	5.12	35.42	167.96	108.73	32.20	30.20	398.00	414.00	379.00	157.00	31.53	27.59	0.00	0.00	383.70	456.28	8.41	4.71	0.00	0.00	0.00	0.00	0.64	0.55	33.83	18.74	4.23	6.46	2.43	0.00	4.54	6.78	0.00	108.55	4.43	0.00
48	2.16	2.49	297.80	69.46	163.01	92.93	19.00	34.80	489.00	161.00	248.00	348.00	911.97	503.76	9.90	3.81	457.37	844.61	5.41	0.00	0.00	0.00	0.00	0.00	0.00	0.00	46.11	56.70	21.50	17.80	0.00	0.00	5.37	4.33	32.54	0.00	1.07	0.81
70	0.92	3.34	4.11	7.74	252.00	631.00	31.50	34.10	612.00	659.00	262.00	497.00	147.00	298.00	2.52	2.06	7932.00	13642.00	13.20	16.10	0.29	1.23	0.50	0.22	2.53	0.69	81.00	153.00	29.00	33.90	4.75	18.80	1.26	2.51	3.38	1.10	7.75	4.27
66	0.46	0.98	6.77	7.83	380.00	231.00	37.10	6.21	1006.00	441.00	1006.00	107.00	56.20	162.00	0.59	0.64	35807.00	19850.00	10.60	14.30	0.10	0.03	0.16	0.06	0.42	0.29	70.00	21.00	9.12	5.17	4.49	1.26	0.36	0.40	5.94	0.14	3.50	2.33
63	0.52	2.10	7.81	8.25	181.00	411.00	5.20	56.90	454.00	911.00	257.00	596.00	199.00	65.30	3.49	0.52	41365.00	48365.00	18.00	13.30	0.06	0.30	0.13	0.32	0.15	0.36	38.40	90.50	8.19	15.70	1.18	5.32	0.07	0.76	34.40	7.59	2.89	3.12
70	10.13	10.13	9.97	9.70	225.00	140.00	36.30	86.00	864.00	1310.00	200.00	692.00	110.00	153.00	1.00	0.38	189.98	291.80	11.90	11.90	0.60	0.23	0.53	0.40	1.09	0.19	15.30	11.60	12.90	11.70	4.26	2.22	0.95	0.24	6.07	3.79	4.76	1.07
79	2.75	6.56	5.01	6.70	312.00	407.00	44.40	60.60	744.00	670.00	849.00	1788.00	36.40	71.30	1.14	3.76	1233.07	2064.50	9.50	21.30	0.29	0.36	0.43	0.75	0.67	0.70	9.13	20.60	17.30	21.90	2.47	5.89	0.75	2.79	11.70	42.00	4.20	4.99
50	1.80	1.80	6.94	/	1164.00	/	115.00	/	130.00	/	274.00	/	60.20	/	0.85	/	1534.00	/	10.30	/	0.48	/	0.45	/	0.18	/	376.00	/	97.70	/	22.80	/	2.28	/	26.10	/	13.80	/

IL, interleukin; TGF, transforming growth factor; TNF, tumor necrosis factor; IFN, interferon; CCL, (C-C motif) ligand. PFS, progression free survival; OS, overall survival. Age is expressed in years. PFS and OS are expressed in months.
